# The Developing Utility of Zebrafish Models for Cognitive Enhancers Research

**DOI:** 10.2174/157015912803217323

**Published:** 2012-09

**Authors:** Adam Michael Stewart, Allan V Kalueff

**Affiliations:** 1 Brain-Body Center, Department of Psychiatry, University of Illinois at Chicago, 1601 W. Taylor Ave., Chicago, IL 60612, USA; 2 Department of Pharmacology and Neuroscience Program, Zebrafish Neuroscience Research Consortium, Tulane University Medical School, SL-83, 1430 Tulane Avenue, New Orleans, LA 70112, USA; 3 ZENEREI Institute, 309 Palmer Court, Slidell, LA 70458, USA

**Keywords:** Cognition, cognitive enhancers, drug screening, neurobehavioral disorders, neuroprotective agents, zebrafish.

## Abstract

Whereas cognitive impairment is a common symptom in multiple brain disorders, predictive and high-throughput animal models of cognition and behavior are becoming increasingly important in the field of translational neuroscience research. In particular, reliable models of the cognitive deficits characteristic of numerous neurobehavioral disorders such as Alzheimer’s disease and schizophrenia have become a significant focus of investigation. While rodents have traditionally been used to study cognitive phenotypes, zebrafish (*Danio rerio*) are gaining popularity as an excellent model to complement current translational neuroscience research. Here we discuss recent advances in pharmacological and genetic approaches using zebrafish models to study cognitive impairments and to discover novel cognitive enhancers and neuroprotective mechanisms.

## 1 INTRODUCTION

Cognitive impairment is a common symptom in multiple brain disorders, including Alzheimer’s disease (AD), Huntingdon’s disease, autism and schizophrenia [1-4]. With the growing global elderly population, and cognitive decline observed with age, there is an urgent need for effective treatments of age- and neurodegenerative-related cognitive deficits [5]. With the limited spectrum of therapies currently available, and the general lack of model systems that fully recapitulate pathogenesis, predictive and high-throughput animal models become particularly important in the field of cognitive research [6, 7].

While rodents have traditionally been used to study cognitive phenotypes [8-12], expanding the range of experimental domains and animal models is recognized as an important strategy of translational neuroscience research [13]. Although modeling neurobehavioral and cognitive disorders has primarily employed rodents in tests of learning, memory and attention [8, 14-16], non-mammalian vertebrates and invertebrates such as *Drosophila melanogaster*, *Caenorhabditis elegans* and several fish species, have emerged as “complementary models” for such research [8].

Due to their complex nervous system and elaborate behavioral repertoire, zebrafish (*Danio rerio*) are gaining popularity as an excellent intermediate model between *in vitro* and *in-vivo* mammalian models for drug screening [7]. Drug discovery has traditionally utilized systemic, target-based screening in purified proteins or cells as primary screens, with *in vivo* models as tertiary screens after more mechanistic cell assays. Although *in vitro* screens have been successful in identifying molecules affecting known mechanisms, there is still the need to identify modulators of complex *in vivo* phenotypes in the whole organism for less well understood pathways – especially those occurring in a physiological and/or pathophysiological context. From this point of view, zebrafish offer a significantly higher throughput *in vivo* screening for phenotypic endpoints compared to other animal models [7, 17].

Furthermore, zebrafish possess robust cognitive abilities, including learning and memory. For example, both associative and non-associative learning has been extensively tested in zebrafish, including cue-based and spatial learning as well as long-term and short-term memory [18-22]. Habituation, extensively investigated in animal models as an evolutionarily conserved adaptive behavior relevant to cognition [22-25], is also being increasingly assessed in zebrafish, as researchers continue to decipher the complexity of fish behaviors [21, 22, 26]. Notably, the habituation response in zebrafish can be modulated through various pharmacological manipulations, and is either attenuated or abolished depending on the receptor class targeted by the drug [26]. Memory has also been well studied in zebrafish, with the animals demonstrating the ability to recall a spatial alternation task for up to 10 days after testing [27]. Importantly, zebrafish cognition is also subject to normal aging processes, as conditioned responses to spatial, visual and temporal cues (associated with changes in cognitive responses to emotionally positive and negative experiences) show reduced generalization of adaptive associations, increased stereotypic and reduced exploratory behavior, and altered temporal entrainment over the course of the zebrafish lifespan [28, 29].

Since the genome and genetic pathways controlling signal transduction and development are highly conserved between zebrafish and humans [30], various molecular tools available render zebrafish particularly useful for determining the mechanisms of action of various classes of psychoactive drugs [31]. Zebrafish models of brain disorders underlying cognitive deficits can also be useful to screen potentially neuroprotective or nootropic compounds for drug development, revealing the mechanisms crucial for new therapeutic treatments [31]. Collectively, this suggests that zebrafish represent a useful model in cognitive neuroscience research [7, 8, 17, 31].

With its small size and transparent larvae, the zebrafish also provides an ideal system to apply novel tools for imaging targeted subsets of neurons and manipulating their activity [32]. Their utility in the recently developed field of *optogenetics* is increasingly allowing researchers to link neural activity with behavior and detect biological events at the cellular level [33-37]. These novel approaches foster the ability to predict how changes in circuit function may lead to altered cognition and behavior relevant to neurobehavioral disorders [38]. Here we discuss recent advances in pharmacological, genetic and optogenetic approaches using zebrafish models to study cognitive impairments and search for novel cognitive enhancers.

## 2 BEHAVIORAL MODELS

Numerous assays have been developed to study zebrafish cognition (Table **1**). For example, the three-chambered tank assesses spatial and nonspatial escape and avoidance discrimination learning, in which the choice behavior and response latency of zebrafish can be measured in the context of spatial and nonspatial discrimination [39]. The roles of motivation and fear/anxiety-like behavior in cognitive processes like learning and memory have been particularly well characterized in zebrafish, with both positive and negative reinforcement demonstrated in the conditioned place preference (CPP) and aversion paradigms [28, 40, 41]. In the CPP test, zebrafish (like rodents) show a preference for an environment that has previously been associated with a substance (e.g., drug or food reward), thus indicating the positive-reinforcing qualities of that substance [40]. The CPP has recently gained recognition for its use studying the cognitive enhancing effects of nootropic drugs in zebrafish. For example, we have shown that chronic exposure to the nootropic drug piracetam significantly improves fish performance in the cued learning test [41], similar to the nootropic effects seen in humans [42, 43] and rodents [44, 45]. Briefly, we used a cued-learning plus-maze test, which consisted of a transparent, four-armed, plus-shaped maze with each individual arm and a central square. One arm was randomly designated as the target arm using a custom-made gel bait as a reinforcement stimulus. To evoke cued learning, a red plastic cue card was placed adjacent to the reward arm, which was randomly changed to prevent bias. In order to evaluate the potential nootropic effects of piracetam, behavioral quantification was performed for the following endpoints: latency to the target arm, the number of target arms, incorrect arm and total arm entries, as well as the duration in the target or incorrect arms (see [41, 46] for further background and protocol details).

The sensitivity of cognitive and several behavioral phenotypes to drugs such as piracetam supports the utility of zebrafish in developing novel screens for compounds with potential cognitive enhancing or neuroprotective properties [41]. For example, modafinil has been shown to improve attention for well-rested individuals, while maintaining wakefulness, memory and executive functions [47, 48]. However, while modafinil has similar effects on the sleep-wake cycle in larval zebrafish as it does in mammals (e.g., increasing wakefulness by lengthening wake-bouts) [49], the potential cognitive enhancing effects of this drug have not yet been investigated in aquatic models, and merit further scrutiny. Other potential drugs of interest include phenotropil, meclophenoxate, semax, oxiracetam, nefiracetam and aniracetam, which also exert nootropic effects in rodents [50-52], and may be active in other model species as well.

Similar to rodents [53-55], evidence of higher cognitive functions in zebrafish suggests that key loci (and, most likely, cognitive maps) can also be created by fish [56-58]. In rodents, homebases serve as “safe” home sites to which animals repeatedly return after exploring the environment, as well as strategic “reference points” to orient and organize their exploration [53]. Recently, the ability to establish such homebases has been reported in zebrafish, suggesting that this behavior can be sensitive to various agents modulating cognitive functions [56, 57]. In line with this prediction, our pilot studies testing the psychotropic hallucinogenic drug ibogaine [59] showed a striking increase in zebrafish homebase behavior following acute 20-min ibogaine exposure (which is, perhaps, not surprising given the ability of ibogaine to modulate cognitions in humans and rodents [60-62]). We have also recently shown that zebrafish, like rodents, scale their locomotor activity depending on the size of the tank and exhibit an inherent behavioral organization in a new environment [63]. Collectively, these findings provide viable assays for measuring altered cognition in fish during their exploration of novel environments. 

The utility of zebrafish to study age-related impairments in cognitive function has also recently been recognized. Compared to other models such as *C. elegans*, *Drosophila* and mice, the relatively long life span (3–5 years) of zebrafish offer a distinct advantage for aging research, providing a model of very gradual rather than rapid decline in specific physiological functions and systems, similar to that observed in humans [28, 64]. In line with this, several behavioral models have been established to assess zebrafish cognition within the aging process, such as the degree of place preference or avoidance, latency to initiating exploration, and choice between the arms within the T-maze [28, 65]. Notably, the age-related changes associated with zebrafish behavior and cognition appears to be subject to genetic and environmental factors. For example, increases in actelylcholine levels delay the onset of age-related changes in cognition, whereas the genotoxic stress of gamma-irradiation accelerates cognitive decline [28]. 

Of considerable importance is the extension of zebrafish behavioral assays to modeling the cognitive and behavior phenotypes arising from specific neurobehavioral disorders. Schizophrenia, for example, is characterized by a multitude of positive and negative symptoms, including social withdrawal as well as cognitive deficits [66]. Mice have traditionally been used to study these behavioral abnormalities, including deficits in working memory, sensory motor gating and hyperlocomotion [67-69]. Pre-pulse inhibition (PPI) is also often employed in mouse to establish the relevance of a model to schizophrenia, examine gene function and test potential therapeutics [69]. As an evolutionarily advantageous response (providing protection from dangers in the natural habitat), the startle response in adult and larval zebrafish can be modulated by weak prepulses in a manner similar to mammalian PPI. For example, administration of the dopamine agonist apomorphine to larval zebrafish has recently been shown to suppress PPI of the startle response [70]. Importantly, the ability to study the modulation of PPI in the zebrafish startle response may provide the opportunity to define the neural circuitry underlying a basic form of behavioral regulation and offer insights into neurobehavioral disorders such as schizophrenia [7, 70]. Importantly, zebrafish may be well suited for other assays commonly used to examine cognitive processes relevant to schizophrenia. In particular, whereas schizophrenic patients show deficits in 2-D object recognition tasks, the novel object recognition (NOR) test (similar to the visual paired-comparison task in humans) [71, 72] has been widely utilized in rodent models of schizophrenia [73-75]. Given that zebrafish display overt behaviors related to novel object inspection, including altered approach latency, freezing and shoaling [76], extension of the NOR test to zebrafish models of schizophrenia may be a promising avenue of investigation.

## 3 OTHER PHARMACOLOGICAL MODELS

The investigation of novel drug targets for treating cognitive impairments associated with neurological and psychiatric disorders is an important focus of neurobehavioral research. Many promising therapies are progressing through preclinical and clinical development, and offer the potential of improved treatment options for cognitive impairments in AD and schizophrenia. The use of both adult and larval zebrafish in large scale, high-throughput screens for various psychotropic drugs has been extensively validated [77-79]. Given the sensitivity of zebrafish behavior and cognition to various pharmacological manipulations [78, 80], they represent a promising novel organism to study the effects of psychotropic compounds, such as those that may attenuate cognitive impairment in models of neurological disorders. 

The cognitive deficits observed in aging and neurodegenerative disorders (e.g., AD) have been linked to decreased cholinergic transmission [81-84]. While acetylcholinesterase (AChE) inhibitors are currently the first line treatment for AD [85, 86], they have been less promising therapeutically, producing only modest improvements in cognitive function with undesirable side effects [87-89]. Thus, agents which increase the release of acetylcholine by direct activation of presynaptic nicotinic acetylcholine receptors (nAChRs) have been suggested as a potential alternative [90]. For example, nicotinic agonists (including nicotine) improve memory function in multiple cognitive assays in rodents and monkeys, while impairments have been frequently reported following treatment with nicotinic antagonists [91-94]. The pro-cognitive effects of nicotine have been established in several animal models [91, 92, 94], and the genetic linkage between nAChRs and several cognitive deficiencies has also been established clinically [95, 96]. Overall, the importance of nAChR subtypes in modulating cognitive processes has been extensively validated, suggesting that activation of nAChR by selective nAChR ligands may be a viable approach to enhance cognitive performance. With the growing support for neuronal nAChRs as valid pharmacological targets, zebrafish have been recently used as a model system to study the role of specific nAChR subtypes [97, 98]. Notably, the zebrafish genome contains the complete complement of human neural nAChR subunit genes. For 8 of the 12 human genes, exactly one zebrafish ortholog exists, while for the 4 remaining human genes, two zebrafish homologs have been identified [99, 100]. Thus, while zebrafish have been used to assess the effects of nicotine on cognitive function [39, 101, 102], their role in studying nAChRs as putative drug targets is becoming particularly promising.

While nAChRs have been implicated extensively in human and animal studies of attention, learning and memory, the α4ß2 and α7 nicotinic receptor subunits (comprising the majority of the nicotinic receptor subtypes within the brain) are heavily involved in cognitive functioning [103]. Notably, both nicotinic α7 and α4β2 receptors have recently been implicated in the nicotinic anxiolysis in zebrafish, and are significantly attenuated by either α7 or α4β2 antagonists [104].

Evidence suggests that nAChRs may contribute to cognitive function by acting in local circuits of the hippocampus and cerebral cortex. For example, in the rodent hippocampus, α7-containing nAChRs are located postsynaptically on inter-neurons (where they mediate fast cholinergic excitatory synaptic transmission) and presynaptically (where their activation increases intra-terminal Ca^2+^ levels and facilitates glutamate release) [105-107]. The hippocampus receives afferent projections, and contains a wide distribution or α7 nAChR receptors, thereby rendering it an ideal circuit to study the effects of α7 in *sensory gating*. In particular, deficits in PPI in sensory gating have been shown in schizophrenic patients, and serve as a primary means of assessing higher-order processing known to be involved in cognitive functioning. [108-110]. Moreover, PPI is also capable of being modulated in rodents as a function of nAChR activation [111-113]. In line with this, modulation of PPI in the zebrafish startle response may also be possible, similar to the response observed following dopamine agonist administration [70].

Similar to the effects demonstrated in humans [114-116] and rodents [117-119], antipsychotic drugs suppress PPI disruptions evoked in the zebrafish startle test [70]. However, whereas antipsychotic drugs are widely used for the treatment of psychotic symptoms in patients with schizophrenia, *typical* (first-generation) antipsychotics often lead to severe motor side effects due to the blockade of dopamine D_2_ receptors [120]. Alternatively, *atypical* (second-generation) antipsychotics are increasingly used in the treatment of schizophrenia [121]. Atypical antipsychotics, although less potent in blocking central D_2_ receptors, have affinity for a wide range of other receptors including dopaminergic D_1_ and D_4_, serotonergic 5-HT_2A_, 5-HT_6 _and 5-HT_7_, adrenergic α_1_, histaminergic H_1_, and muscarinergic M_1_ [122, 123]. In line with this, acute exposure to the NMDA antagonist dizocilpine (MK-801) induces psychoses-like deficits in cognition and social interaction, which can be reversed by acute administration of atypical, but not typical, antipsychotics [124]. Zebrafish demonstrate high sensitivity to MK-801-induced psychotic-like effects, which are also attenuated in this aquatic model by antipsychotics [125-127].

Finally, while the main focus of this paper is on the use of zebrafish for testing cognitive enhancers, this sensitive animal model can also be used to study the opposite aspect – screening drugs that impair memory. Indeed, several studies have shown the sensitivity of zebrafish to ketamine, MK-801, scopolamine, and ethanol all known to disrupt memory [128-131]. Therefore, the possibility of using zebrafish-based screens to test memory-inhibiting drugs also seems promising.

## 4 GENETIC MODELS

The ease of genetic manipulations and the robust behaviors of zebrafish make them particularly amenable to functional and genetic dissection [132]. Early development of the zebrafish nervous system has been well characterized, presenting an effective model for studying development and disease processes in the nervous system [133]. Notably, morpholino antisense oligonucleotides (MO), mRNA or transgenes all alter gene expression and can be observed in the transparent zebrafish embryo. MOs can be designed to block translation of a particular gene or to block the splicing of particular exons into transcripts. Over-expression studies can also be performed by injecting sense mRNA of a gene. Finally, transgenic zebrafish can also be developed by using efficient vectors (e.g., the “sleeping beauty” transposase system) to insert genes under the control of tissue-specific promoters [134].

Importantly, zebrafish are increasingly utilized to investigate the molecular mechanisms underlying several brain disorders. For example, AD pathogenesis occurs through several pathways, with AD brain characterized by amyloid beta (Aβ) peptide deposition leading to amyloid plaques and neurofibrillary tangles (NFT) as well as aberrant presenilins. Three genes clinically implicated in AD pathogenesis are *APP*, *presenilin 1* (*PS1*) and *presenilin 2* (*PS2*), as their mutations have been identified in cases of familial AD [135]. In line with this, mice lacking the orthologues *Psen1* and *Psen2* in their forebrain show multiple phenotypes resembling AD [136]. Similarly, orthologues of *PS1* and *PS2 *have also been identified in zebrafish (*psen1* and *psen2*), demonstrating a highly conserved alignment in primary structure with human proteins. Such conservation has allowed for *in vivo* analysis, using homozygous null *psen1* mutants displaying decreased cell proliferation and neurogenesis [134]. Since presenilin proteins are ubiquitously expressed, transgenic expression of mutated *psen1* genes in zebrafish lacking endogenous *psen1* activity can further elucidate the effects on *psen1 *function of mutations causing AD [137].

Morpholino antisense methods have been applied in zebrafish to examine potential cognitive and neurological impairment that may arise from subtle abnormalities in brain development due to alterations in the functions of candidate susceptibility genes. In particular, susceptibility genes underlying the pathogenesis of schizophrenia, such as Disrupted-in-schizophrenia 1 (DISC1) and Neuregulin 1 (NRG1), are increasingly being examined within zebrafish to provide novel insights into their role in brain development [138]. Whereas mouse models with mutations in *Disc1* have behavioral and pathological alterations characteristic of schizophrenia [139, 140], efforts to characterize the expression profiles of these key genes have revealed a similar development and functionality to their mammalian homologues [138]. Additionally, morpholino technology has recently been utilized in a zebrafish model of attention-deficit/hyperactivity disorder (ADHD) [141, 142]. For example, the disruption of Latrophilin 3 (LPHN3) gene function produces a hyperactive/impulsive motor phenotype which can be reversed by ADHD drugs such as methylphenidate and atomoxetine [142]. Overall, significant utility of morpholino technology lies in its ability to correlate gene activity and behavioral phenotypes with the development of the brain circuitry.

## 5 NOVEL TECHNIQUES AND METHODS 

In parallel with pharmacological and genomic modeling of cognitive impairment, mounting evidence shows the feasibility of detecting and tracking these diseases *via *disease-specific morphological signatures [143]. For example, clinical imaging studies have linked AD to brain atrophy [144, 145] and Parkinson’s disease to decreased grey matter [146, 147]. In line with this, the application of bioimaging techniques to several animal models and the development of digital brain atlases are revealing the bidirectional relationships between functional connectivity and disease pathology such as neurodegeneration [143]. Such *connectome*-based analyses in conjunction with digital atlas technology further increases our understanding of how cognitive processes emerge from their morphological substrates, and how these processes are affected when this structural substrate is disrupted [148].

With the growing utilization of zebrafish in neuroscience research, a population-based atlas of the zebrafish brain has recently been established, serving as a digital reference for anatomical localization comprising delineations based upon multiple magnetic resonance sequences and histological stains [143]. This publically available atlas provides important information on the spatial relationships of neuronal structures, as well as quantitative information and an accurate stereotaxic coordinate system. The combination of voxel-based comparisons and parametric comparisons for individual regions may also allow the effects of genetic and/or environmental manipulation on the anatomical phenotype to be assessed [143]. Recently, digital atlas data has also been further integrated with the visualization of gene expression data to allow researchers to identify spatiotemporal and quantitative aspects of gene expression in different stages of zebrafish development [149].

The use of *connectome*-based analyses helps to comprehensively map the neural connections from the level of single neurons and synapses (microscale) to the level of anatomically distinct brain regions and inter-regional pathways (macroscale) [148, 150-152]. While currently focused on the human brain, *connectome*-based approaches may be further extended to animal models of neuropathology [152]. For example, the use of Brainbow imaging techniques has successfully revealed the connectivity between different neural populations in mice and *Drosophila* [153-155]. With rapid advances in the fields of optical microscopy and fluorescent dyes, further incentive to use an optically accessible model such as the zebrafish is supported. As zebrafish have fewer neurons than mice, more extensive networks of connectivity between different brain regions may be determined, also reflecting other highly dynamic processes, such as axon growth and pruning, which may be used as potential biomarkers of cognitive functions [156].

With the advent of new genetically encoded optical tools, researchers have recently begun to directly manipulate neuronal firing, probe neuronal-circuit function and control behavior on milliseconds timescales. The emergence of optogenetics has enabled monitoring and control of neuronal activity with minimal perturbation and unprecedented spatiotemporal resolution [157-161]. Briefly, light-gated channels and pumps allow the activation and silencing of neurons, while fluorescent proteins further allow for the sensing of calcium or membrane potential. Importantly, such optogenetic tools provide significant opportunities for analyzing neural circuits, especially those involved in cognitive and behavioral disorders [158, 161, 162]. For example, circuit-level perturbations in the brain's electrical activity may underlie cognitive deficits in schizophrenia and autism – disorders hypothesized to arise from hyperexcitation within neural microcircuitry. However, direct investigation of cell- and circuit-level effects of changes in cellular excitation has been precluded, as cell-specific pharmacological agents are lacking, and homeostatic processes can occur downstream of synaptic and intrinsic excitability alterations. Emerging optogenetics techniques have recently begun to bridge this gap, having been applied to study altered cellular excitation as well as quantify the effects on information transmission network activity in freely moving animals [162].

Zebrafish offer an ideal system to apply optical techniques for imaging the nervous system, as well as genetically encoded tools for targeting subsets of neurons and manipulating their activity [32]. Notably, its small size allows all neurons from a defined circuit to be monitored at once under a laser scanning microscope. For example, photoconvertible proteins can define and image circuits as well as link zebrafish behaviors with neuronal development, such as in developing spinal circuitry controlling tail movements in larvae [163]. Optical reporters of neuronal activity have also been useful for mapping brain regions with specific topographic arrangements in zebrafish, such as mapping the olfactory bulb in explants from adult zebrafish during their responses to odors [164]. Furthermore, numerous studies have applied optogenetic techniques to link neural activity with behavior in zebrafish. Specifically, optogenetic tools have been used in zebrafish to detect biological events at the cellular level, such as voltage spikes, increased intracellular Ca^2 +^, and the fusion of neurotransmitter-bearing vesicles [33-35, 37].

Since the first optogenetic studies in zebrafish several years ago [33-35, 37], there has been a great expansion in the number of protein tools used, circuits targeted, and behaviors analyzed. Continued improvement of optogenetic tools in terms of the sensitivity and temporal control of manipulations should lead to the development of novel and better behavioral assays to further aid in linking patterns of neural activity to their corresponding behaviors [37]. Moreover, optogenetic approaches may help predict how changes in circuit function alter cognition and behavior, elucidating how circuit-level manipulations may ultimately be used for the treatment of neurobehavioral disorders [38].

## 6 CONCLUSION 

The utilization of diverse and novel models, such as the zebrafish, may foster a better understanding of the complex actions of cognitive enhancing or neuroprotective agents, eventually leading to more effective treatments for various cognitive and affective brain disorders. The sensitivity of some zebrafish memory-related behaviors to nootropic compounds further supports their utility in developing novel screens for compounds with potential cognitive enhancer properties. Additionally, the use of various mutant or transgenic zebrafish may enable further characterization of genetic and physiological mechanisms involved in learning and memory, as well as in fish sensitivity to cognitive enhancing or neuroprotective drugs.

Continued advances in optogenetics, high-resolution *in vivo *imaging, and bioinformatics techniques have created new opportunities to obtain a greater understanding of higher brain functions under healthy and diseased conditions. Recent studies have highlighted the potential of zebrafish for detailed measurements of neuronal activity patterns, manipulations of defined cell types *in vivo,* and for studies of causal relationships between circuit function and behavior. The small brain of zebrafish may also be exploited in future research for the reconstruction of neuronal wiring diagrams by 3D electron microscopy [165]. The exhaustive and quantitative analysis of neuronal activity and connectivity will eventually be important to derive principles governing neuronal circuit function by at both the theoretical and practical levels, especially in relation to disease pathogenesis. In light of their relatively complex cognition and elaborate behavioral repertoire, such circuit analyses is not limited to simple systems and should be applied to neural pathways underlying higher order processing relevant to cognitive deficits [166].

## Figures and Tables

**Table 1.** Selected Behavioral Assays Used for Assessing Cognitive Function in Zebrafish

Task	Application	References
Inter- and intra-trial habituation	An adaptive behavior that can be used to assess short-term and longer-term learning and memory	[21, 22, 26]
Spatial alternation task	Evaluation of spatial learning and memory function	[27]
Three-chambered tank	Assesses spatial and nonspatial escape and avoidance discrimination learning,	[39]
Aversion and conditioned place preference (CPP) paradigms (e.g., T-maze)	Assesses positive and negative reinforcement; can be coupled with a cue-based stimulus to evaluate learning	[28, 40, 41]
Latency to initiating exploration	Assessed age-related changes in cognitive function	[28, 40, 41, 65]
Novel object recognition	Reflects cognitive processes involved in inspection and recognition of novel object	[76]
Homebase formation	Reflects spatial memory and space recognition in zebrafish	[56, 57]
Pre-pulse inhibition (PPI)	Evaluates modulation of the startle response, relevant to neurological disorders such as schizophrenia	[70]
